# Perillyl Alcohol and Its Drug-Conjugated Derivatives as Potential Novel Methods of Treating Brain Metastases

**DOI:** 10.3390/ijms17091463

**Published:** 2016-09-02

**Authors:** Thomas C. Chen, Clovis O. Da Fonseca, Axel H. Schönthal

**Affiliations:** 1 Department of Neurological Surgery, Keck School of Medicine, University of Southern California, Los Angeles, CA 90089, USA; thomas.chen@medmail.usc.edu; 2 Department of General and Specialized Surgery, Antonio Pedro University Hospital, Fluminense Federal University, Niterói, RJ 24220, Brazil; clovis.orlando@uol.com.br; 3 Department of Molecular Microbiology & Immunology; Keck School of Medicine, University of Southern California, Los Angeles, CA 90089, USA

**Keywords:** blood-brain barrier, brain drug delivery, intranasal, metastasis, perillyl alcohol

## Abstract

Metastasis to the central nervous system remains difficult to treat, and such patients are faced with a dismal prognosis. The blood-brain barrier (BBB), despite being partially compromised within malignant lesions in the brain, still retains much of its barrier function and prevents most chemotherapeutic agents from effectively reaching the tumor cells. Here, we review some of the recent developments aimed at overcoming this obstacle in order to more effectively deliver chemotherapeutic agents to the intracranial tumor site. These advances include intranasal delivery to achieve direct nose-to-brain transport of anticancer agents and covalent modification of existing drugs to support enhanced penetration of the BBB. In both of these areas, use of the natural product perillyl alcohol, a monoterpene with anticancer properties, contributed to promising new results, which will be discussed here.

## 1. Introduction

The precise incidence and prevalence of brain metastasis is not known for several reasons. Epidemiologic studies are oftentimes based on tumor registries, hospital records or death certificates [1,2], but these sources usually are incomplete; while the primary cancer is always recorded, the presence of intracranial lesions oftentimes is not noted. In addition, brain metastatic spread can be asymptomatic and remain unrecognized; or it is symptomatic, but intentionally ignored in patients severely ill with progressive primary disease; or it might simply not be mentioned in patients’ discharge papers [3]. Routine central nervous system (CNS) imaging to look for brain lesions is not recommended in patients with advanced cancer who lack symptoms of brain involvement [4]. Pure population-based studies have concluded incidence rates ranging from 8.3 to 14.3 per 100,000 population, suggesting up to 46,000 new cases in the United States (USA) each year [5]. However, historical data based on autopsy studies suggested early on that, for certain types of cancers, the incidence of brain metastases might be significantly higher than what was suggested by epidemiological studies [6,7].

To more precisely define incidence rates, Gavrilovic and Posner [3] and, independently, Nayak et al. [5] performed meta-analyses that took all available variables into account and considered all types of brain metastases from different primary tumor types, based on studies in the USA and several other countries. Although neither group calculated specific numbers, both reports concluded that the above stated numbers represent a significant underestimation of true brain cancer incidence. In an effort to further enumerate the magnitude of this condition and anticipate its current and future burden, Davis et al. [8] pursued the unusual approach of calculating the expected number of metastatic brain tumors that would subsequently develop among incident cancer cases for one diagnosis year, based on USA cancer incidence data from the year 2007. Their study findings conservatively estimated that about 70,000 patients with newly-diagnosed cancer in 2007 would be expected to develop brain metastases over their remaining lifetime.

Brain metastases commonly present as one of two major types: parenchymal or leptomeningeal [9]. Leptomeningeal metastasis (oftentimes referred to as leptomeningeal carcinomatosis (LMC)) represents secondary tumor growth that develops in the microenvironment containing the cerebrospinal fluid (CSF) and the linings of the brain. Initial seeding is thought to be accomplished mainly by tumor cells arriving from the systemic circulation and crossing the blood-CSF barrier into the CSF [10,11]. In comparison, parenchymal brain metastasis (PBM) is secondary tumor growth in the essential and distinctive tissue of the brain after tumor cells have entered the brain parenchyma via the blood-brain barrier (BBB) [10,12]. PBM is more common than LMC, although both types may occur in the same patient. The five most common sources of PBM and LMC are lung, melanoma, breast, kidney and colorectum [3,13]. Median survival time after diagnosis of LMC is generally significantly shorter than after diagnosis of PBM [14,15]. For the purposes of this review, we will not explicitly distinguish between PBM and LMC, but will refer to brain metastases in general. It should be noted, however, that certain aspects of diagnosis and treatment may differ between PBM and LMC; the interested reader is referred to recently published LMC-specific review articles for additional details [15,16,17].

Regrettably, therapeutic approaches for patients with brain metastases are limited, and these patients inevitably face a dismal prognosis. Treatment options oftentimes are restricted to cranial radiotherapy and surgical resection, although the latter oftentimes is inadequate because the majority of patients present with multiple brain lesions when brain involvement is first detected [18,19], and many such patients receive only palliative care [9,20,21]. Whole-brain radiotherapy comes at the expense of neurological toxicity and other side effects, which can include nausea, severe headaches and dementia. In cases of three or fewer intracerebral metastatic sites, stereotactic radiosurgery may provide some benefit [22,23]; although the general side effects of this particular approach are usually limited, they can occasionally be more serious [24].

Advancements in chemotherapeutic agents have provided significant hope to people diagnosed with many types of extracranial cancers; however, their success in fighting malignant brain lesions has been quite limited. Even temozolomide, the widely-used standard of chemotherapeutic care for malignant glioma, extends the survival of glioblastoma patients by only 2.5 months [25]. When studied in patients with brain metastases, temozolomide yielded no, or at best modest, benefit [26,27,28], consistent with its lack of activity in mouse tumor models with established breast metastases in their brains [29].

The major obstacle preventing effective chemotherapy of brain metastases is the BBB [30]. This barrier prevents brain entry of most drugs, despite indications that its barrier function might be variably compromised within brain tumor lesions, depending on the cancer type [31]. Conventional cancer drugs, such as etoposide, cisplatin, capecitabine, vincristine, methotrexate, bleomycin or cyclophosphamide, do not effectively penetrate the BBB, although some of them may achieve occasional therapeutic responses when given at higher than normal dosages [32,33,34].

A recent detailed analysis [35] of 2000 individual lesions of breast-brain metastases in mice demonstrated great heterogeneity between individual lesions, and even within the same lesion, with regard to BBB permeability. Although 90% of these lesions showed BBB compromise, their uptake of paclitaxel or doxorubicin was less than 15% of that of other tissues or peripheral metastases. Paclitaxel and doxorubicin reached cytotoxic concentrations only in a small subset (~10%) of the most permeable brain metastases. Therefore, despite being partially compromised within brain tumor lesions, the BBB is still operational enough to substantially impede effective drug delivery and spoil most therapeutic activity. Notably, these considerations apply to temozolomide, as well: despite the often-repeated claim that this malignant glioma drug penetrates the BBB well, its concentration in cerebrospinal fluid is only 20% of that measured in the plasma of patients [36]. Thus, chemotherapeutic regimens for brain-metastatic cancers, as well as for primary brain malignancies, continue to remain in need of substantial improvements [37,38,39].

It is quite conceivable that improved delivery methods for chemotherapeutic drugs and targeted anticancer agents could achieve superior outcomes in patients with malignant brain lesions, and a number of strategies are being explored to increase brain targeting of drugs. These include invasive interventions, like intraparenchymal, intraventricular and intrathecal delivery [40,41], or non-invasive techniques, like intranasal drug delivery, or drug modifications that include prodrug approaches and the conjugation of drugs with various ligands or antibodies [42,43,44,45]. Preclinical and clinical studies with the natural compound perillyl alcohol have provided evidence that this monoterpene might be particularly suited for the purposes of intranasal drug delivery, as well as for the modification of existing drugs toward enhanced BBB penetration. In the following, we will present these new developments and discuss the value of perillyl alcohol for the purposes of the therapy of intracranial malignancies.

## 2. Intranasal Drug Delivery

Traditionally, the nasal route has been used to deliver drugs to treat local diseases, such as nasal infections, allergies, rhinorrhea or sinusitis. Subsequently, this mode of delivery was also applied as an alternative to parenteral injections for the systemic delivery of various compounds, including peptides and small proteins, as well as a variety of low molecular weight drugs. Intranasal drug uptake provides several advantageous characteristics. The nasal mucosa features a large surface area with extensive vascularization, overall vigorous blood flow and decreased levels of enzymes as compared to the digestive system and the liver. Combined, these elements facilitate direct access, quick absorption of drugs and the avoidance of first-pass hepatic metabolism, thereby providing for greater bioavailability and rapid onset of drug response [46,47,48,49,50].

One of the intensely-investigated aspects of intranasal delivery is the objective to deliver pharmaceutical agents directly from the nose to the brain. Direct nose-to-brain transport is based on the idea that this route of drug delivery would circumvent, rather than cross, the BBB, resulting in enhanced access of drugs to the intracranial lesion. It thus may offer an attractive alternative, or addition, to drug delivery via the systemic circulation across the BBB, in particular for those drugs with little or no BBB penetration. Altogether, the combination of direct nose-to-brain transport together with mucosal uptake is envisioned to further support accelerated drug uptake, greater bioavailability, as well as rapid onset of drug action in the brain [46,47,48,49,50].

**Figure 1:**
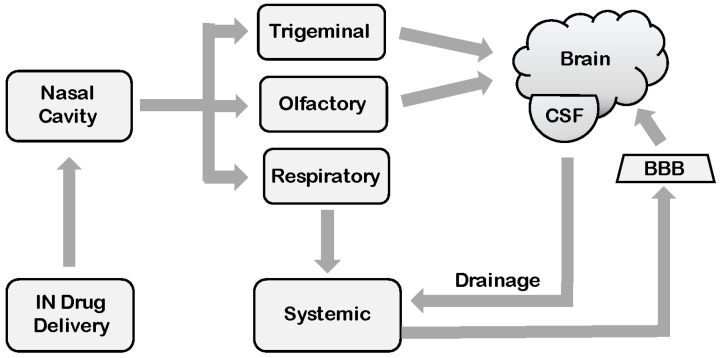
Pathways for reaching the brain after intranasal (IN) delivery. Following drug administration into the nasal cavity, three different pathways are available for potential transport to the brain: (i) uptake by the respiratory epithelium into the systemic circulation, followed by brain delivery across the blood-brain barrier (BBB) (if the compound is BBB permeable); (ii) uptake by the olfactory epithelium and transfer via the olfactory bulb into the brain parenchyma; (iii) uptake by the trigeminal network and transfer into the brain (diagram modeled after [57]). CSF, cerebrospinal fluid.

Although the exact mechanisms, as well as overall efficacy, of direct nose-to-brain transport are still a matter of controversy [51,52], there is accumulating evidence that this principle could become a valuable addition to cancer therapeutic regimens [46,49,53,54,55]. Based on anatomical features, distinct direct and indirect pathways are available, which potentially could be utilized by intranasally-delivered drugs to enter brain and CSF. Besides blood vasculature and the lymphatic system, the olfactory pathway or the trigeminal nerve pathway may become involved as the most direct gateways to the brain [47,53,54,55,56] (Figure 1).

### 2.1. Olfactory Pathway

The olfactory region in humans involves less than 10% of the nasal cavity. The sensory fibers of the olfactory bulb represent the only anatomical extension of the central nervous system that is physically exposed to the environment. The olfactory pathway begins at the neuronal olfactory receptors within the olfactory mucosa. From there, olfactory neurons project through the cribriform plate, which partitions the nasal cavity from the cranial cavity, to mitral cells within the bulbus olfactorius. Next in this pathway, the olfactory bulbs connect to different regions of the brain, which include the olfactory tract, the anterior olfactory nucleus, the piriform cortex, the entorhinal cortex, the amygdala and the hypothalamus. Several lines of studies indicated that upon intranasal drug delivery, intraneural and also perineural transport can take place along these projections [54,58,59,60,61].

When a drug travels from the olfactory region toward the brain parenchyma or CSF, it needs to pass through the olfactory epithelium and possibly also the arachnoid membrane surrounding the subarachnoid space. As detailed by Pardeshi and Belgamwar [56], there are three separate potential passages crossing the olfactory epithelium: (i) a transcellular pathway that may involve endocytosis for somewhat less lipophilic drugs or passive diffusion for highly lipophilic compounds; (ii) a paracellular pathway primarily for hydrophilic drugs; and (iii) the olfactory nerve pathway, which takes up drugs into the neuronal cells by endocytosis or pinocytosis and subsequently carries them by intracellular axonal movement toward the olfactory bulb. Altogether, these various processes of transport involve such diverse mechanisms as carrier-mediated transport, efflux transport, transcytosis, paracellular passive diffusion and transcellular passive diffusion [56].

### 2.2. Trigeminal Nerve Pathway

The trigeminal nerve is the largest of the cranial nerves, featuring three main branches: (i) the ophthalmic nerve (V1); (ii) the maxillary nerve (V2); and (iii) the mandibular nerve (V3); which together provide sensory information from the nasal cavity, oral cavity, eyelids and cornea to the brain. Only V1 and V2 innervate different areas of the nasal passages through the ethmoidal, nasopalatine and nasal extensions and, therefore, represent those branches of the trigeminus that are important for nose-to-brain drug delivery [56,57,58,61,62].

A study by Thorne et al. [63] was the first to demonstrate trigeminus-mediated brain delivery of radiolabeled insulin-like growth factor-I (IGF-I) after intranasal delivery to rats. This method of delivery activated IGF-1 signaling pathways in the brain, confirming that at least portions of the IGF-1 reached CNS target sites intact. The determination of gamma counting of microdissected tissues and high-resolution phosphor imaging of tissue sections indicated two quick pathways of brain entry: one was the peripheral olfactory system connecting the nasal passages with the olfactory bulbs and rostral brain regions, and the other was the peripheral trigeminal system connecting the nasal passages with brainstem and spinal cord regions [63]. A carefully-executed study by Johnson et al. [64] used an intranasally-applied infrared dye that reached the brain within 10 min, and distribution of the dye was deemed consistent with it being transported by the trigeminal nerve.

While some details underlying the transport of drugs from the nose to the brain are not yet completely characterized, it is likely that a combination of different routes is responsible, although specific pathways may predominate, depending on the physiochemical properties of the therapeutic agent [53].

### 2.3. Intranasal Delivery of Cancer Drugs

A number of therapeutic agents, including small molecules and macromolecules, have been used to target the olfactory system. For example, intranasal insulin-like growth factor 1 (IGF1), erythropoietin and deferoxamine showed protection against stroke in animal models; neuroprotective peptide NAPVSIPQ (also called NAP) was used to treat neurodegeneration; and intranasal insulin improved memory and functioning in patients with Alzheimer’s disease [65,66,67,68,69,70,71]. Other components that are being investigated for intranasal delivery to the brain include genes encoding neurotrophic factors, viral vectors, small interfering RNAs (siRNAs), lysosomal enzyme, leptin and stem cells [72,73,74,75,76,77,78]. In several of these studies, no concomitant increases in serum concentrations were noted after intranasal delivery, supporting the prevalence of direct nose-to-brain transport [59,74,77,79,80,81]. Altogether, while the intranasal route of drug delivery has been extensively investigated in the context of neurodegeneration and general brain function, this modality has received less attention with regard to the problem of intracranial tumor growth [82,83].

In the context of intracranial tumor chemotherapy, early in vivo studies of methotrexate (MTX), raltitrexed and 5-fluorouracil in rats demonstrated that these antineoplastic agents could be directly transported into the CNS through the olfactory pathway, resulting in significantly greater brain exposure than intravenous dosing [80,84,85,86]. In experiments investigating drug effects on intracranial tumor growth, MTX (combined with oral acetazolamide, an inhibitor of the secretion of cerebrospinal fluid [85]) was administered either via the intranasal (IN) route or via intraperitoneal (IP) injection to rats with 9L glioma cells implanted in their brains [87]. The drug was given three times at two-day intervals, and after 10 days, the brains were collected and the tumors weighed. It was found that tumor weight from IP MTX-treated rats was only minimally lower (by 20%) than that from untreated rats, in keeping with the known difficulty [88] of MTX to effectively cross the BBB. In comparison, IN MTX was significantly more effective and resulted in substantially (80%) reduced tumor burden. The average final tumor weight from untreated control rats, IP-injected rats and IN-treated rats was 300, 240 and 60 mg, respectively. These differences were statistically highly significant between the control and IN groups (*p* < 0.001) and also between the IP and IN groups (*p* < 0.001), clearly highlighting the therapeutic benefit of intranasal delivery in the case of MTX [87].

A study by Hashizume et al. [89] reported successful intranasal delivery of a telomerase inhibitor to athymic rats harboring intracerebral human U251 glioblastoma cells. GRN163, a thio-phosphoramidate-based oligonucleotide with the ability to block telomerase function, was administered intranasally daily over a three-week period, and the survival of animals was recorded. In these experiments, rats that remained untreated, or were treated with a mismatch control oligo, showed a median survival of 35 days, whereas survival of GRN163-treated animals was greatly extended to 75 days [89]. In another study, using rats with intracranially-implanted C6 glioma cells, Taki et al. [90] demonstrated a small, yet statistically-significant survival benefit of intranasal camptothecin (CPT), a chemotherapeutic topoisomerase I inhibitor. Intriguingly, delivery of CPT within nano-sized micelles, spiked with a cell-penetrating peptide, further enhanced the therapeutic benefit of this intranasal approach. The authors concluded that such modified nanoparticles might support efficient drug penetration of the nasal epithelia, overall leading to increased drug uptake [90]. Related efforts aimed at increasing intranasal drug uptake by a variety of modifications and formulation strategies are ongoing [53,58,91].

### 2.4. Intranasal Delivery of Perillyl Alcohol

In the context of attacking intracranial malignancy via intranasal drug delivery, perillyl alcohol (POH) is the only compound so far that has undergone testing and validation in clinical trials. POH is a natural monocyclic terpene derived from limonene and the mevalonate pathway in certain plants, such as citrus, peppermint, spearmint, lavender and lilac oils, sage, celery, cherries and others [92]. When administered orally to different types of xenograft mouse or rat tumor models, it revealed potent activity against different types of cancer, including those of the breast, liver and pancreas [93,94,95,96,97]. In the chicken chorioallantoic membrane (CAM) model, POH blocked the migration of C6 rat glioma cells [98]. In a mouse model with orthotopically-implanted breast cancer cells, intraperitoneal injection of POH prevented spread from the primary tumor site to the regional lymph nodes [97]. This latter study is of particular interest, because invasion of the lymph nodes represents a key step of early metastatic spread in breast cancer. In this study, 75 mg/kg POH were administered via IP injection three times a week over six weeks to nude mice with orthotopically-implanted human KPL-1 breast cancer cells. At the end of the treatment period, the average tumor weight in POH-treated animals was 36% smaller than that of untreated mice, and this difference was statistically significant (*p* < 0.05). With regard to the invasion of the axillary lymph nodes by tumor cells, it was described that none of the POH-treated animals (*n* = 13) were positive, but three of 15 (20%) untreated animals presented with lymph node invasion [97]. Although the results of statistical analysis (*p* value) of this latter observation were not provided, this preliminary outcome points to the possibility that POH might be able to impact these early steps of metastatic spread. However, further studies to validate this aspect are urgently needed.

Based on the collective outcome of the above-mentioned animal studies, POH was formulated in soft gelatin capsules and tested in a number of phase I and II cancer trials, where it was given orally three to four times each day for several months [99,100,101,102,103,104,105]. However, the results were disappointing. Because a high dosage was needed (gram quantities), patients had to swallow a large number of capsules, which caused unrelenting intestinal side effects (nausea, satiety, eructation, vomiting) and fatigue. Although toxicities were fairly mild to moderate, some patients found the unremitting nature of the intestinal problems hard to tolerate and withdrew from treatment [101]. In addition, contrary to what had been documented in experimental animal tumor models, the antitumor activity of POH in patients was unconvincing. As a result, oral POH as an approach to cancer therapy was abandoned [106].

In an effort to harness the cancer therapeutic potential of POH, yet avoid its dose-limiting intestinal problems, intranasal delivery was explored as an alternative to oral dosing. Using mice with intracranially-implanted, drug-resistant human glioblastoma cells, Cho et al. [107] administered POH into the nostrils of animals once every other day. The highest dosage applied was only 1.9 mg/kg, which is substantially lower than what was commonly used in earlier in vivo experiments with oral gavage or intraperitoneal injections of POH [93,94,95]. The results showed extended survival of POH-treated animals, clearly demonstrating the therapeutic efficacy of intranasal POH. In addition, these investigators noted reduced invasive capacity of POH-treated tumor cells, further pointing to the possibility that this compound might also have antimetastatic activity [107]. However, as in vitro invasion assays investigate only one step of the metastatic cascade, additional studies are required to establish whether or not POH indeed harbors anti-metastatic potential.

**Figure 2:**
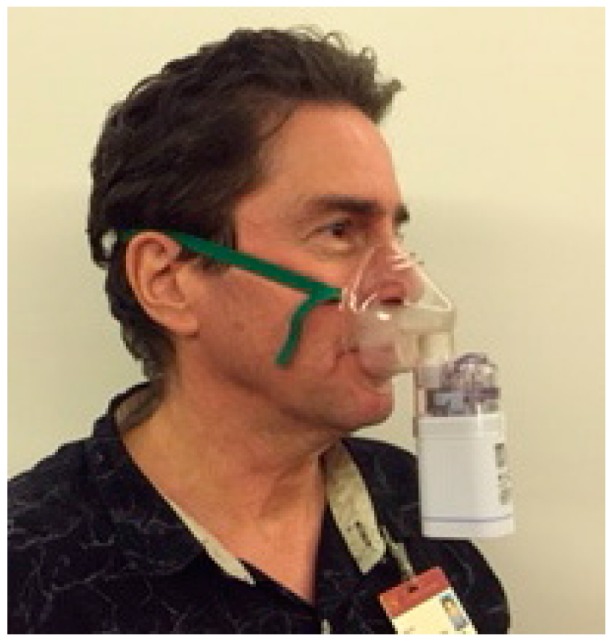
Example of intranasal (IN) anticancer drug delivery device. A large number of different models of nasal spray pumps and pressurized aerosol inhalers are available and in use worldwide [91]. Shown here is an example of a nebulizer that is being used to deliver perillyl alcohol (0.3% *v*/*v*) to patients with brain cancer. The device can be filled (23 drops of perillyl alcohol diluted in 3 mL of mineral water [111]) and operated by the patients themselves. It performs hands-free and is powered by a rechargeable battery.

Clinical studies performed in Brazil explored intranasal POH as an option for patients with recurrent glioblastoma. Standard initial therapy for glioblastoma patients consists of surgery, followed by radiation and chemotherapy with temozolomide [108]. Despite this tripartite effort, the tumors recur in the vast majority of cases, leaving patients without effective treatment options and spelling dismal prognosis. Using a cohort of 37 such recurrent patients, an initial phase I/II study [109] administered POH at 55 mg per dose, four times a day (totaling 220 mg per day), via intranasal delivery. Patients self-administered POH with a lightweight, portable, battery-operated nebulizer device (Figure 2). The ease of administration, in combination with the lack of serious side effects, resulted in very high patient compliance (reported as >95% [110]). In addition, this delivery strategy produced encouraging clinical outcomes (see the details below).

Two follow-up reports [110,112] presented updates on these studies that eventually included 155 patients with recurrent glioblastoma (GBM), 27 with grade III astrocytoma (AA) and 16 with anaplastic oligodendroglioma (AO), as well as escalated POH doses up to 133 mg (532 mg total per day). This regimen continued to be well tolerated and effective, with 19% of patients remaining in clinical remission after four years of continuous, exclusive POH treatment. Some of the data were compared to historical control groups: for example, patients with recurrent GBM treated with intranasal POH showed 5.9 months of survival advantage compared with 2.3 months of mean survival of the control group that only received supportive treatment (*p* < 0.0001). It was noted that the overall survival benefit of intranasal POH was greater in recurrent patients with secondary GBM (i.e., where GBM had developed from lower-grade glioma) than in patients with primary (de novo) GBM; also, tumor location in the basal ganglia appeared to respond somewhat more favorably to POH than tumors at other brain locations. The underlying reason for these differences are unclear and remain to be elucidated.

Of note, the four times daily intranasal treatment regimen appeared to be well tolerated. It was reported that the side effects “were almost nonexistent, even in patients treated for over 4 years” [112]. POH occasionally caused nose soreness, but rarely nosebleed. All in all, the authors concluded that very long-term intranasal delivery of POH represents a safe, non-invasive and effective strategy for patients with otherwise treatment-resistant glioma [110,112]. Based on the promising results of these Brazilian studies, a similar clinical trial with recurrent grade IV glioma patients has been initiated in the United States (NCT02704858).

It appears that POH represents the only intranasally-applied cancer therapeutic agent tested in the clinic so far. While current studies are focusing on primary malignant brain cancer, there are indications that this regimen might also be active against brain metastases secondary to systemic cancer types. As detailed above, in preclinical models, POH has revealed anticancer potency against a variety of different tumor types. Although its potential anti-metastatic activity so far has not been thoroughly established, the few available studies do provide limited evidence to support this expectation. Furthermore, animal models and clinical studies provided proof-of-principle that intranasal delivery results in intracranial activity and circumvents the intestinal toxicity that had been dose limiting after oral dosing. Therefore, there is good rationale for expanding these studies to include the analysis of intranasal POH for the therapy of brain-metastatic cancers.

## 3. Perillyl Alcohol-Drug Conjugation for Improved Blood-Brain Barrier (BBB) Penetration

While the above discourse presented intranasal drug delivery as a means to circumvent the obstacle posed by the BBB, other research efforts are aimed at modifying the chemical structure of cancer therapeutic agents in order to force their penetration through the BBB. Intriguingly, it appears that here, as well, POH harbors exciting features that may lead to increased brain entry and enhanced intracranial activity of partner drugs.

Designing new drugs, or modifying existing drugs, to increase their likelihood of penetrating the BBB represents a considerable challenge. It generally requires a balance between optimizing the pharmacokinetic and physiochemical characteristics and making the best compromises in properties critical for drug activity and delivery. This enormous complexity will not be reviewed here, as appropriate reviews are available [12,113,114,115]. Instead, we will select and present an illustrative example, involving POH, which is providing promising leads toward improved BBB penetration of appropriately-modified chemotherapeutic agents.

### 3.1. In Silico Prediction of BBB Penetration

A number of determinants are being considered in an effort to predict how effectively a given compound might cross the BBB, and available computer models are programmed to integrate these determinants to formulate such predictions [115,116,117,118]. For example, the BBB Penetration Module software (Advanced Chemistry Development, Inc.; ACD/Labs, Toronto, ON, Canada) is specifically designed to analyze a given chemical structure and to predict whether it is permeable enough to exhibit activity in the CNS. This module provides predictions of both the rate and extent of BBB permeation (expressed as Log*PS* and Log*BB* constants, respectively) and allows the ranking of compounds according to their passive transport across the BBB. Log*PS* values are calculated using physicochemical property values, such as lipophilicity (Log*P*) and ionization (pKa) as inputs. Calculated Log*BB* values represent the steady-state distribution ratio between brain tissue and plasma. Also considered is the brain/plasma equilibration rate (Log*PS* × *fu*, *brain*), where *fu*, *brain* is the fraction of drug that is unbound, i.e., pharmacologically active, in brain tissue. A detailed discussion of this modeling approach and the predictive value of these different parameters can be found in the literature [117,119]. The program provides a qualitative estimate as to whether brain uptake is sufficient for CNS activity and whether compounds are likely to undergo transport across the BBB by mechanisms other than passive diffusion. At the end of the analysis, the program visualizes the position of a test compound on a plot of BBB parameters, in comparison to a large number of well-characterized CNS drugs and peripheral drugs.

**Figure 3:**
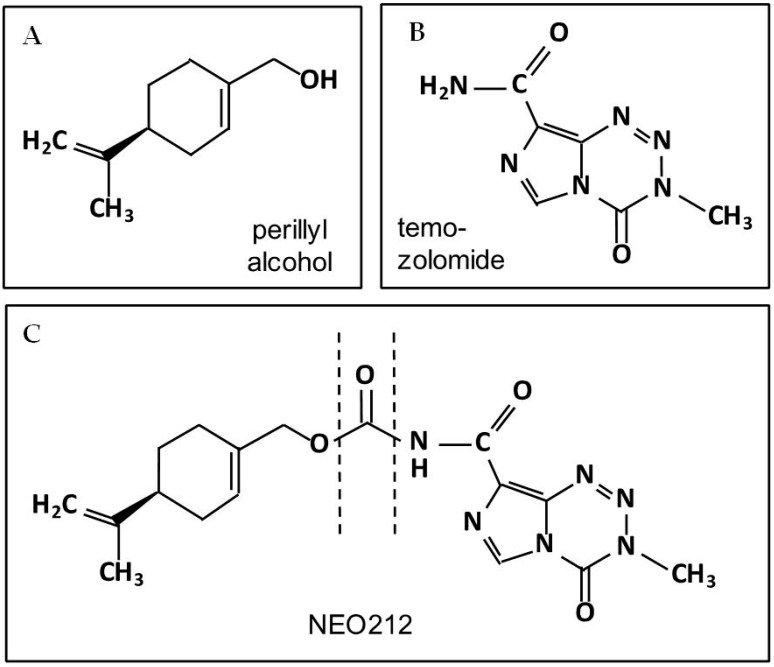
Chemical Structures. Shown are the chemical structures of (**A**) the monoterpene perillyl alcohol and (**B**) the imidazotetrazine temozolomide; (**C**) shows NEO212 (perillyl alcohol conjugated to temozolomide), with the two components covalently conjugated via a carbamate bridge (between dashed lines).

**Figure 4:**
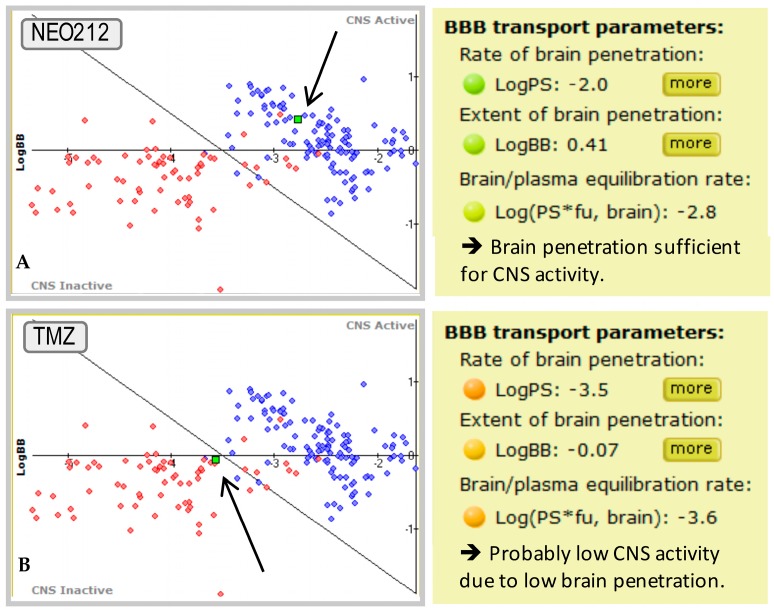
Central nervous system (CNS) activity plot using ACD/Lab’s BBB Penetration Module software (Advanced Chemistry Development, Inc.; Toronto, ON, Canada). Arrows point to the test compound (green dot) in each panel, i.e., NEO212 (**A**) and temozolomide (TMZ, **B**), respectively. Blue dots represent 73 well-characterized CNS-active drugs. Red dots represent 62 known peripherally-acting drugs with little or no brain activity. As shown, NEO212 (**A**) is located well within the cluster of brain-active compounds, whereas TMZ (**B**) is located borderline; in agreement with its known ability to only suboptimally (20%) penetrate the BBB. *Y*-axis: Log*BB*; *X*-axis: Log*PS* × *fu, brain*. Note the colored circles in the yellow data squares to the right: green color highlights values that are supportive of brain activity, orange signifies borderline values, and red color indicates values that are not supportive of brain activity.

In our own efforts to discover improved brain-permeable cancer therapeutics, we used ACD Labs’ software to investigate the covalent conjugation of POH to known anticancer drugs. This analysis yielded several positive hits, where the addition of POH resulted in increased predicted brain activity of the respective novel fusion compound. One of these chimeric compounds, POH conjugated to temozolomide (called TMZ-POH or NEO212; Figure 3), yielded remarkably favorable values and predicted superior brain activity, whereas in comparison, the unmodified temozolomide molecule produced only borderline values (Figure 4). This latter finding is in keeping with observations stated earlier: despite being the standard of chemotherapeutic care for malignant gliomas, temozolomide extends median survival of glioblastoma patients by only 2.5 months [25]; when studied in patients with brain metastases, it yielded insignificant benefit [27,120,121,122,123,124,125]; and in an animal model of brain-metastatic breast cancer, it was inactive against well-established brain lesions [29]. Pharmacologic measurements indeed confirmed that temozolomide enters the brain sub-optimally: only 20% of serum levels can be measured in the cerebrospinal fluid [36], which in retrospect is not entirely surprising, as temozolomide was not specifically developed or optimized as a brain-targeting agent.

### 3.2. Preclinical Validation of In Silico Prediction

In silico analysis predicted that covalent conjugation of POH to a number of established, FDA-approved drugs would result in their enhanced penetration of the BBB and increased intracerebral activity. To provide proof-of-principle that this computer prediction was valid, one of the novel POH fusion compounds, NEO212, was studied in preclinical tumor models.

Two separate reports provided evidence that NEO212 is highly active against intracranial tumor growth. In one of these studies [126], temozolomide-resistant U251 glioblastoma cells were implanted into the brains of mice, followed by treatment with vehicle control, temozolomide or NEO212. The results showed significantly prolonged survival of those animals receiving NEO212, as compared to vehicle control or temozolomide treatment. In the other study [127], intracranially-implanted triple-negative MDA-MB-231 cells were used as a breast cancer brain metastasis model. Here, as well, NEO212 displayed striking therapeutic activity. While temozolomide treatment resulted in a small extension of median survival (six days longer than the vehicle treatment), NEO212 was able to extend survival by 28 days and, thus, was 367% more effective than temozolomide in this model [127]. In related studies in mice, it was confirmed that brain entry of NEO212 was significantly greater than entry of temozolomide [128].

Altogether, NEO212 serves as a first-in-class compound where brain entry and the intracranial therapeutic efficacy of an existing pharmaceutical agent is being enhanced via covalent conjugation to POH. It is conceivable that this type of modification is applicable not only to cancer drugs, but also to other compounds, where currently-achievable BBB penetration is sub-optimal and in need of improvement.

## 4. Conclusions and Outlook

Over the past decade, better control of systemic neoplastic disease has resulted in prolonged survival of patients with advanced cancers. In combination with earlier detection of metastatic spread to the brain, however, these advances have led to progressively increasing prevalence of patients with brain metastasis, which threatens to compromise gains made in systemic therapy. The chemotherapeutic treatment of brain metastases is severely impeded by the presence of the BBB, which minimizes effective access of most cancer drugs to the sites of intracerebral lesions. Consequently, it is essential to find novel approaches to more effectively penetrate, or perhaps entirely circumvent, this significant obstacle.

In this context, we have presented examples of new approaches, derived from the experience with glioblastoma patients, which might become applicable to brain-metastatic patients, as well. Intranasal delivery of POH has yielded encouraging results in patients with primary brain cancer, and it is not unreasonable to rationalize that it should yield similar outcomes in patients with brain metastases. However, while clinical trials with intranasal POH have been initiated for recurrent glioblastoma patients in the United States, no such studies are currently being planned for patients with metastatic brain lesions.

From the large field of drug modifications aimed at the enhancement of BBB penetration, we presented NEO212, where temozolomide, a sub-optimally brain-targeting molecule, was conjugated to POH. Based on in silico prediction, as well as preclinical brain tumor and brain metastasis models, NEO212 appears to be a novel compound with great therapeutic promise. However, the validation of this prediction has to await testing of NEO212 in clinical trials, which have not yet started. Furthermore, based on the success of the intranasal delivery of POH, it might be intriguing to determine whether brain-targeted activity of the conjugated NEO212 compound could be increased even further via intranasal delivery, as well.

## Abbreviations

BBB: blood-brain barrier; CNS: central nervous system; CSF: cerebrospinal fluid; IN: intranasal; LMC: leptomeningeal carcinomatosis; NEO212: perillyl alcohol conjugated to temozolomide; PBM: parenchymal brain metastasis; POH: perillyl alcohol; TMZ: temozolomide.
